# Navigating CDC recognition for the National DPP in socially vulnerable communities: barriers, facilitators, and recommendations

**DOI:** 10.1186/s13690-025-01532-4

**Published:** 2025-03-03

**Authors:** Taynara Formagini, Ariba Rezwan, Daphnee Rodriguez, Maya Venkataramani, Matthew James O’Brien, Elva Arredondo, Boon Peng Ng

**Affiliations:** 1 Department of Family Medicine, University of California San Diego, 9500 Gilman Dr. La Jolla, San Diego, CA 92093 USA; 2 School of Social Work, University of Central Florida, Orlando, FL USA; 3 Institute for Behavioral and Community Health (IBACH), San Diego State University, San Diego, CA USA; 4 Keck School of Medicine, University of Southern California, Los Angeles, CA USA; 5 Division of General Internal Medicine, Johns Hopkins University, Baltimore, MD USA; 6 Department of Medicine, Division of General Internal Medicine and Geriatrics, Northwestern University Feinberg School of Medicine, Chicago, IL USA; 7 Department of Psychology, San Diego State University, San Diego, CA USA; 8 College of Nursing, University of Central Florida, Orlando, FL USA; 9 Disability, Aging, and Technology Cluster, University of Central Florida, Orlando, FL USA; 10 Department of Population Health Sciences, College of Medicine, University of Central Florida, Orlando, FL USA

**Keywords:** National Diabetes Prevention Program (National DPP), CDC recognition, Socially vulnerable communities, Barriers and facilitators

## Abstract

**Background:**

The CDC National Diabetes Prevention Program (National DPP) lifestyle change program is a nationwide initiative to prevent or delay the onset of type 2 diabetes in adults with prediabetes. The CDC recognition status (i.e., pending, preliminary, full, or full-plus) signifies that a program meets specific quality, fidelity, and effectiveness standards. However, organizations—especially those serving socially vulnerable communities —often face significant challenges in achieving and maintaining this recognition. We aimed to explore the barriers and facilitators related to achieving and maintaining CDC recognition among organizations delivering the National DPP in socially vulnerable communities.

**Methods:**

This qualitative descriptive study used a web-based questionnaire to gather insights from 27 organizations delivering the National DPP in socially vulnerable communities. Respondents shared their experiences regarding challenges in attaining and maintaining CDC recognition, strategies to overcome these challenges, and recommendations for CDC support. Thematic analysis was conducted to identify and report emerging themes.

**Results:**

Funding availability, strong partnerships with community organizations, and flexible program delivery models were identified as key facilitators for achieving and maintaining CDC recognition. Major barriers included difficulties with participant recruitment and retention as well as insufficient funding to support program delivery costs. Respondents recommended increasing flexibility in recognition requirements, advocating for better reimbursement models, expanding training opportunities, and promoting collaboration between delivery organizations to enhance sustainability.

**Conclusion:**

Our study highlights key factors influencing the achievement and maintenance of CDC recognition for delivering the National DPP lifestyle change program among organizations in socially vulnerable communities. Addressing these factors through flexible program requirements (e.g., risk-adjusted models), improved funding models, strengthened support from the CDC, and collaboration between organizations could improve program sustainability.

**Supplementary Information:**

The online version contains supplementary material available at 10.1186/s13690-025-01532-4.

**Table:** 

Text box 1. Contributions to the literature
• This study identifies barriers and facilitators to achieving and maintaining recognition for delivering the National Diabetes Prevention Program (National DPP), a key initiative to prevent diabetes across the U.S.
• It highlights funding availability, strong community partnerships, and flexible delivery models as facilitators, while recruitment challenges, retention issues, and insufficient funding remain significant barriers.
• Recommendations to better support organizations offering the National DPP include flexible recognition requirements, risk-adjusted reimbursement models, and increased funding to support program delivery and sustainability.
• This work contributes actionable insights for policy and practice to improve the long-term sustainability and equity of the National DPP nationwide.

## Introduction

Prediabetes affects 38% of the U.S. adult population, with up to 50% progressing to type 2 diabetes (hereafter referred to as diabetes) within five years if no preventive measures are taken [1, 2]. However, evidence indicates that the onset of diabetes can be delayed or prevented through healthier behaviors and participation in structured, evidence-based lifestyle change programs. The landmark U.S. Diabetes Prevention Program (DPP) randomized controlled trial and subsequent translational studies have demonstrated the feasibility and effectiveness of such interventions in reducing diabetes risk in real-world settings [3–5]. These interventions are designed to promote modest weight loss (5–7%) and moderate physical activity through year-long lifestyle change programs among individuals with prediabetes.

In response to these findings, the Centers for Disease Control and Prevention (CDC) launched the National Diabetes Prevention Program (National DPP) in 2012, aiming to scale these lifestyle change interventions nationally and mitigate the growing burden of diabetes in the U.S [6]. The National DPP is a large-scale public health initiative designed to deliver a standardized, evidence-based curriculum nationwide. Led by the CDC, the program is implemented in collaboration with community and healthcare organizations, which deliver the lifestyle change program. The program includes 16 weekly sessions during the first six months, followed by a minimum of six monthly sessions in the latter half of the year [7].

To ensure program quality and fidelity, the CDC conducts periodic evaluations of organizations implementing the National DPP and grants these organizations CDC recognition status (i.e., pending, preliminary, full, or full-plus) [8]. These assessments verify adherence to the program structure and standards, ensuring alignment with the behavioral intervention principles demonstrated in the DPP studies. Moreover, the CDC monitors program outcomes to evaluate their effectiveness in reducing diabetes risk [8]. To achieve and maintain CDC recognition, organizations need to follow an approved curriculum, meet participant recruitment and retention targets, submit biannual data reports, and meet program outcomes. For example, full recognition requires that at least 60% of participants achieve 5% weight loss or 0.2% HbA1c reduction. Alternatively, full recognition can be achieved by documenting 4% weight loss along with: (1) attending at least 17 sessions; or (2) attending 8 sessions while averaging 150 min of physical activity per week [8]. Once organizations receive recognition from the CDC, they become eligible for reimbursement from Medicare, Medicaid in some states, and a growing number of commercial health insurance plans if they become suppliers for these entities [9]. While reimbursement can, to a certain extent, support organizational funding to sustain program delivery, not all organizations can achieve or maintain CDC recognition [10, 11]. Approximately 18% of CDC-recognized organizations have voluntarily discontinued their participation in the National DPP between 2012 and 2019 [12], and disparities in CDC recognition status among organizations in socially vulnerable communities have been recently reported [13]. Although participant enrollment in the program has increased since 2012, a report published in 2022 showed that enrollment rates may be leveling off [12]. This is particularly relevant in socially vulnerable communities as previous research has indicated a lack of program availability in counties marked by high socioeconomic disadvantage [14–16].

Together, this information offers a broad overview of the National DPP’s accomplishments and challenges to date, but little is still known about the factors associated with achieving and maintaining CDC recognition to deliver the program. To address this knowledge gap, our study explored barriers and facilitators in achieving and maintaining CDC recognition among organizations delivering the National DPP lifestyle change program. Understanding these factors can help National DPP suppliers anticipate success factors and challenges, identify potential solutions, and inform practice, ultimately enhancing the implementation and sustainability of the National DPP and similar programs in the United States.

## Methods

### Design and setting

This study used an exploratory, qualitative, descriptive design and was conducted from September 2022 to February 2023, using a web-based, cross-sectional questionnaire through the Qualtrics Survey Software. We received approval from the University of Central Florida Institutional Review Board [STUDY00004472]. The Standard for Reporting Qualitative Research (SRQR) was used to ensure transparency in reporting our methods [17].

### Sampling strategy and participants

Purposive sampling was used to identify organizations that delivered the National DPP lifestyle change program nationwide [18]. The CDC maintains a publicly available registry of all CDC-recognized organizations with their name, addresses, contact information, recognition status (i.e., pending, preliminary, full, or full-plus), and delivery mode (i.e., in-person, online, distance-learning, combination) [19]. While the list is periodically updated, organizations that have discontinued the program or lost their recognition status may still appear on the registry until updates are made. As a result, we anticipated reaching out to organizations that were actively delivering the program with CDC recognition, those delivering the program without CDC recognition, and some that were no longer actively delivering the program. Inclusion criteria were: (1) staff member of an organization on the registry, (2) ability to read in English, and (3) involved in the National DPP administration or delivery. In addition, we focused on organizations in socially vulnerable communities (i.e., those in high social vulnerability areas based on the CDC/ATSDR Social Vulnerability Index - SVI) [20], as their experiences could provide valuable insights into the program delivery and CDC recognition process, applicable to a range of organizations. The SVI is a composite measure based on 15 social factors grouped into four domains: socioeconomic status, household composition and disability, minority status and language, and housing type and transportation. Higher SVI scores indicate greater social vulnerability, with the CDC categorizing vulnerability levels as Low, Low-Medium, Medium-High, or High at the county level. Detailed descriptions of these domains and factors are available elsewhere [21, 22]. For this study, we identified organizations located in areas classified as High social vulnerability based on their Zip codes.

### Procedures

In September 2022, we downloaded the CDC National Registry of Recognized Diabetes Prevention Programs, which listed 2,218 organizations. To identify those located in socially vulnerable communities, we linked the Zip codes from the CDC Registry with the HUD-USPS ZIP Code Crosswalk file and the CDC/ATSDR 2018 SVI [21, 22]. We then extracted the name and contact information of the 501 organizations meeting these criteria to form the study sample.

Next, we randomly selected organizations from this subset using Excel and utilized the provided contact information to reach out by phone. During the phone calls, we identified ourselves as researchers from the University of Central Florida and asked to speak with a staff member familiar with the National DPP. We explained the study objectives, invited them to complete a questionnaire, and mentioned that participants would be compensated $20 for their time and effort. If they agreed, we obtained their email and sent a secure link to the questionnaire. If they declined, we inquired about the reasons for their decision, which included being busy, the organization no longer having the program, or privacy concerns. When we were unable to reach organizations by phone, we searched for their websites using public web domains to locate email addresses. We then sent detailed study information via email, along with an invitation to participate by completing the questionnaire.

Following qualitative design guidelines, we aimed for 20–25 completed questionnaires, as this sample size is generally considered sufficient to reach code saturation (i.e., the point at which no new themes are expected to emerge from the data) [23]. We contacted 104 organizations. Organizations were called a maximum of three times, resulting in 206 calls. Follow-up emails were sent to those who accepted but had not completed the questionnaire within a week. Of the 36 organizations that agreed to participate, 27 (75%) completed the survey. Respondents consented via an attached document in Qualtrics before accessing the questionnaire. The questionnaire took an average of 25 min to be completed. Respondents received a $20 online gift card after completing the questionnaire.

### Questionnaire design and content

We designed a web-based questionnaire using Qualtrics with open-ended and multiple-choice questions. The questions were tailored to reflect whether the organizations were (a) currently delivering the National DPP lifestyle change program with CDC recognition, (b) delivering the lifestyle change program without CDC recognition, or (c) not currently delivering the program. All three versions addressed similar constructs and experiences, with adjustments in tense and wording (e.g., who delivers the program vs. who used to deliver the program).

Open-ended questions matched the methodological fit of the project, including our research question, prior work, and research design [24]. Given the limited research on organizations’ experiences in achieving and maintaining CDC recognition, we opted for a qualitative approach. The choice of a questionnaire over traditional in-depth interviews was driven by its practical advantages, including allowing participants to respond at their convenience, which facilitated participation by reducing scheduling barriers. Additionally, this format aligned with our study’s objective of pilot testing the use of online questionnaires as a data collection method, with the aim of informing the development and distribution of a multiple-choice questionnaire in future studies.

The questions were developed through a literature review on National DPP implementation and insights from the first and senior author (TF and BPN), given their experience with the program [13, 25]. The questionnaire elicited respondents to provide insights on attaining and maintaining CDC recognition, including challenges encountered by their organizations, strategies employed to overcome these challenges, and suggestions on how the CDC can assist organizations in achieving and maintaining recognition. The full open-ended questionnaire is available in the supplementary material.

Multiple-choice questions were developed to identify the characteristics of the organization and the individuals they served. Respondents were asked about their organizational setting, program modality, curriculum type, and funding coverage methods. Characteristics of the population served included race/ethnicity, language spoken, and age. Respondents were required to answer all questions but could review and change their answers through a back/forward button. The full questionnaire was piloted by two members of the research team (DR and AR) who were not involved in its development, and their feedback was incorporated to refine the final version.

### Data analysis

Following the completion of all respondents, the questionnaire was downloaded and imported into NVivo 12, a software for qualitative analysis [26]. Thematic analysis was used to identify, analyze, and report patterns from the data set, which involves creating themes from the patterns found [27, 28]. The primary author initially coded a subset of four questionnaires, which consisted of taking notes, observations, and interpretations to identify emerging patterns and themes. Then, a codebook was developed, which included the names of these initial patterns and their description and examples. The codebook was used to code the remaining transcripts, allowing for new themes to emerge from the data. The analysis was conducted by the first author and reviewed by the senior author. The findings were organized based on the most significant themes found. The quotes from the respondents are presented with a number ID to facilitate the attribution of quotes. Multiple-choice questions were analyzed with STATA/SE 18.0, and we included median, range, and percentage of responses.

## Results

### Organizational characteristics

Table 1 describes the characteristics of organizations included in the study. A total of 21 organizations were delivering the program with CDC recognition; three were delivering the program but lost their CDC recognition or voluntarily discontinued [#22, #23, and #24], and three were not delivering the program anymore [#25, #26, and #27]. Two-thirds of respondents belonged to either a healthcare (29%) or a community-based organization (37%). Most organizations (63%) employed a combination of delivery methods, incorporating in-person, online, or distance learning. The CDC PreventT2 curriculum was the predominant curriculum, utilized by 85% of the organizations. Respondents reported diverse funding sources to cover the program, including 63% utilizing grants, 55% utilizing organization funds, and 26% receiving reimbursement through health insurance, with many organizations reporting more than one funding source.

**Table 1:** National DPP lifestyle change program: organizations characteristics (*n* = 27)

Organizations’ characteristics	*n* (%)
*Program delivery status*	
Delivering the program with CDC recognition	21(77.8)
Delivering the program without CDC recognition	3 (11.1)
Not delivering the program anymore	3 (11.1)
*Type of setting*	
Community-based setting	8 (29.6)
Hospital/healthcare setting	10 (37.0)
Health departments	4 (14.8)
Faith-based setting	1 (3.7)
University	3 (11.1)
Health plan company	1 (3.7)
*Modality*	
In-person	7 (26.9)
Online (i.e., asynchronous)	1 (3.7)
Distance-learning (i.e., synchronous)	2 (7.4)
Combination of methods of delivery	17 (63.0)
*Program personnel*	
Lay workers (community health workers, health educators/coaches)	11 (40.7)
Specialized healthcare professionals (nurses, physicians, dietitians)	8 (29.6)
A combination of both	6 (22.2)
Other	2 (7.4)
*Type of curriculum*	
PreventT2 curriculum (2021)	23 (85.2)
2102 CDC-developed curriculum	2 (7.4)
Unsure	1 (3.7)
Other	1 (3.7)
*Coverage sources *^a^	
Reimbursement from health insurance	7 (25.9)
The organization covers the costs	15 (55.6)
Participants pay for the program	5 (18.5)
Grants	17 (63.0)
Other	3 (11.1)
*Method to collect program outcome data*	
Using paper	4 (15.4)
Using a computer or tablet (electronic system)	7 (26.9)
A combination of both	15 (57.7)
*Program language delivery*	
English	13 (48.1)
Spanish	3 (11.1)
Both English and Spanish	11 (40.7)

^a^adds to more than 27 because answers were not mutually exclusive

### Characteristics of individuals served

Most respondents noted that the individuals served by their organization represented diverse populations, with 52% reporting a mix of races/ethnicities. Participants served by the organizations reflected a mix of both sexes and a variety of age groups. The median number of participants in the initial session of the program was 12, with a median of 7 participants completing the program (Table 2).

**Table 2:** National DPP lifestyle change program: participants’ characteristics reported at the organizational level

Participants’ characteristics	Distribution
*Race/ethnicity*, n (%)	
Mostly Black non-Hispanic	4 (14.8)
Mostly Hispanic/Latino	9 (33.3)
A mix of races and ethnicities	14 (51.9)
*Sex*, n (%)	
Mostly women	9 (33.3)
A mix of both women and men	18 (66.7)
Mostly men	0
*Age*, n (%)	
Mostly middle-aged adults (40 to 64 years old)	8 (29.6)
Mostly older adults (65 or older)	3 (11.1)
A mix of ages	16 (59.2)
*Income*, n (%)	
Mostly low income	14 (51.9)
Mostly medium income	13 (48.1)
Mostly high income	0
*Number of participants per cohort in the first session of the program*, median (range)	11.5 (1–20)
*Number of participants per cohort complete the program*, ^a^ median (range)	6.5 (1–14)

^a^According to the CDC Diabetes Prevention Recognition Program, participants are considered completers if they attended at least 8 sessions during months 1–6, and the time from the first session held for their cohort to the last session attended by the participant spans at least 9 full months

### Barriers and facilitators influencing the achievement and maintenance of CDC recognition for the National DPP lifestyle change program

This section is organized into four main themes: (I) Factors associated with achieving and maintaining CDC recognition; (II) Challenges in achieving and maintaining CDC recognition; (III) Strategies to address challenges in maintaining recognition; and (IV) Enhancing CDC Support. Below, we summarize the findings for each theme. Two illustrative quotes per theme are presented in Table 3, with additional quotes provided in the supplementary material.

**Table 3:** Themes and selected supporting participants’ quotes

**Theme I. Factors associated with achieving and maintaining CDC recognition**	
Availability of resources and funding sources	*We had strong admin support*,* resources (people) available to work through the process*,* and a team approach between the [university] Health Hub*,* [university] School of Pharmacy*,* and [university] Health System Dietetic Internship in order to kick things off. Grant support through the [state] Department of Health was also imperative. [#5*,* educator*,* dietitian]* *We achieved CDC recognition for NDPP we were able to do so because we had a funding source that allowed us access to resources to engage the community. [#7*,* health education coordinator]*
Team commitment to deliver the program	*Lifestyle Coaches were key. This is a one-year program that requires committed investment in facilitation and follow-up with program participants. It was important for coaches to develop trusting relationships to maintain retention. [#13*,* community health supervisor]* *We had a bilingual team*,* passionate about serving the community. We had CHWs*,* staff and med students. [#24*,* director]*
Partnerships with community organizations	*[the university personnel] have access to local communities with which they work and the skills to set up and deliver programs. [#8*,* community outreach specialist]* *I feel that statewide partnerships were valuable*,* and provided us with access to tools*,* resources*,* and funding that made this possible […]. Working with partners to offload some of the burden of billing allows us to build a case for support for our leadership as well as sustainability at minimal cost to our organization. We are very grateful for the community partners that make this program possible. [#11*,* director of community health]*
**Theme II. Challenges in achieving and maintaining CDC recognition**	
Recruitment and retention of participants	*Challenges we faced in order to achieve recognition were participant retention. Many participants didn’t want to participate in a yearlong DPP program and the few that did would drop after a few weeks of participation […] Recruiting participants and participant retention is a challenge we are still facing in order to maintain the CDC recognition. [#7*,* health education coordinator]* *Challenges our organization encountered when achieving CDC recognition was that sometimes our [participants] had competing interests*,* such as work-related responsibilities*,* which made it difficult for them to attend every class. [#19*,* chronic disease prevention program manager]*
Participants not meeting program outcomes	*We are currently facing challenges in maintaining recognition because we’ve been relying on the weight loss/physical activity standards for recognition*,* but not all participants have been reaching this standard–particularly with the onset of the pandemic and the decrease in physical activity across many populations. [#15*,* MDPP regional coordinator*,* public health educator]* *It was very challenging to meet the weight loss and attendance rates. [#24*,* director]*
Issues around data collection and management	*The data component. We had to ensure we maintained accurate data records. When the standards changed in 2021*,* that extended our Preliminary status*,* and we were unable to receive full recognition. [#13*,* community health supervisor]* *Additionally*,* the database system our state contracts with is oftentimes slow with returning the cleaned data we need to submit for recognition. [#15*,* MDPP regional coordinator*,* public health educator]*
Program costs and financial barriers	*Inadequate reimbursement rates and lack of universal coverage for this program!! We have managed to sustain our programs thanks to grant funding*,* but we won’t be able to do that forever. [#2*,* program director]* *I am not getting full recognition status from Medicare and my organization is not able to receive any payments from various insurance company due to my pending recognition. [#3*,* lifestyle coach]*
**Theme III. Strategies to address challenges in maintaining recognition**	
Additional support to participants to enhance retention	*I put a lot of effort into communicating with participants and made sure to provide as much support as possible to help them maintain their goals for this program. […] When a participant missed a class*,* I made sure to communicate with the person and provided make-up sessions. I also enrolled participants to another national program*,* Walk with Ease*,* in the period of monthly sessions to help participants to stay engaged. These two strategies were pretty helpful. They helped the participants to hold themselves more accountable. [#4*,* program director]* *We increased retention in a few ways-instituted a raffle program where participants received a raffle ticket for each class attended*,* offered individual goal tracking support*,* conducted frequent reminder calls*,* instituted a “buddy” system which increased accountability among participants [#23*,* health equity program director]*
Increasing referrals and recruitment	*I participated in community events to get referrals. It was not successful as the attendees wanted community resources and social needs. [#3*,* lifestyle coach]* *We put a lot of effort into recruitment through our own advertising (Emails*,* flyers*,* community tabling*,* TV*,* radio interviews). We do have some referrals from our hospital*,* and we do get participants from our fitness center*,* which is in the same building as us. [#12*,* DPP coordinator*,* lifestyle coach]*
Partnerships with community organizations	*We are able to maintain recognition thanks to multiple partnerships in the community. We have strong relationships with the local community hospital as well as the local federal Qualified Health Centers. [#7*,* health education coordinator]* *We have great relationships with our state health departments*,* as well as local city/county health care organizations*,* which allows us to combine efforts*,* apply for larger pools of funding*,* and create networks. We take a truly wraparound approach for all participants*,* meeting them where they’re at and helping them overcome challenges. [#11*,* director of community health]*
**Theme IV. Enhancing CDC Support**	
Program adaptations and changes in requirements for recognition	*It would be ideal if the CDC could design a shorter curriculum that can be used in place of the Prevent Type 2 curriculum. There is huge interest in the program but once people learn that its a yearlong program they no longer want to participate. [#7*,* health education coordinator]* *Get rid of the weight requirements for enrollment*,* the weigh-ins*,* and the weight loss requirements. Make it more weight inclusive. Better to go based on [physical activity] minutes and a1c’s to measure health and success. [#9*,* lead outpatient registered dietitian]*
Resources and training	*Continue to provide general resources*,* webinars*,* and training for organizations that are conducting the program. [#4*,* program director]* *Support us with training. Training on the data portion on how to enter*,* what to enter*,* how to access the data. I think a Q&A on a weekly basis may be helpful [#18*,* health education specialist]*
Improving funding and insurance reimbursement models	*Continued funding for these types of programs […] Sustainability is still tenuous - while reimbursements for Medicare/caid have improved*,* relying upon insurance payouts alone would not be sufficient to cover the cost of running the program. [#11*,* director of community health]* *Improve funding models. We were unable to continue the program because of lack of funding. Payment model is inadequate and insufficient. [#24*,* membership director]*
Increasing awareness of prediabetes and of the National DPP	*Promoting the program in the medical community as a resource they can use to help their patients. Promoting HbA1c/blood glucose testing so that more people are aware that they have prediabetes. [#8*,* community outreach specialist]* *Creating further awareness of prediabetes within the healthcare community and building bridges between these types of programs and healthcare providers […] Continuing to innovate in communication about these types of programs to eligible individuals. [#11*,* director of community health]*
CDC is supportive	*We have generally found the CDC to be incredibly supportive*,* particularly from a data review standpoint. [#2*,* program director]* *CDC has been extremely helpful during the process. Their data collection may be tedious*,* but they offer quick and supportive feedback as needed. They have also met with me on an individual basis to review evaluation reports and provide feedback for the next cohort. Maintaining this level of support would be great. [#13*,* community health supervisor]*

## Theme I. factors associated with achieving and maintaining CDC recognition

Respondents highlighted three main factors associated with CDC recognition: (1) the availability of resources and funding sources to cover the program costs; (2) having team members committed to delivering the program; and (3) partnerships with other community organizations.

### Availability of resources and funding sources

Respondents highlighted that funding from local and state health departments, the healthcare system, or the university was important in achieving recognition to offer the program [#4, #5, #7, #10]. Collaboration between different sectors within the same institution to provide administrative support and resources was highlighted by a health educator [#5]. Overcoming barriers, such as covering staff time costs, was described by a director of community health, who emphasized the role of grant funding in minimizing this obstacle [#11].

### Team commitment to deliver the program

Team commitment emerged prominently in our analysis: Respondents mostly highlighted the importance of certified lifestyle coaches, who played a crucial role in recruiting and retaining participants in the program [#7, #9, #10, #12, #13, #24, #25]. This commitment extended beyond professional qualifications to the development of trusting relationships with participants [#13]. The involvement of a bilingual team in delivering non-English language National DPP was described as a key component of program delivery [#24].

### Partnerships with community organizations

Partnerships with community organizations emerged as another key factor in the analysis. One respondent cited that those partnerships enhanced access to tools, resources, and funding, which reduced the burden for the organization [#11]. A program director illustrated the development of an initiative that enabled their organization to deliver the program in collaboration with faith-based organizations [#6]. Additionally, a community outreach specialist emphasized their partnership with university personnel, who provided access to additional participants [#8].

## Theme II. Challenges in achieving and maintaining CDC recognition

Respondents provided a series of challenges they faced: (1) difficulties in the recruitment and retention of the required number of participants in each cohort; (2) difficulties faced by participants in meeting program outcomes required for full recognition (i.e., participants not meeting weight loss and/or physical activity goals); (3) issues around participant data collection and reporting; and (4) financial barriers around program costs.

### Recruitment and retention of participants

Referral difficulties and challenges in recruiting National DPP participants were commonly cited [#1, #3, #7, #8, #16, #22, #26]. Retention of participants, which is also a requirement for achievement and maintenance of program recognition status, was also challenging. A common issue was the decline in participation rates as the program progressed [#4, #5, #6, #17], with some respondents expressing the difficulty of retaining individuals throughout the year-long program [#7, #27]. Respondents noted that social barriers impacted participant retention [#2, #11], including transportation and access to affordable healthy food [#2]. While program staff often tried to proactively address these barriers, doing so was time-consuming. [#11].

### Participants not meeting program outcomes

Respondents also highlighted difficulties related to participants’ achievement of the outcomes required for maintaining full recognition and/or receiving insurance reimbursement [#1 #4, #14, #15, #23, #24, #27]. Achieving weight loss requirements was mentioned as a key challenge by respondents [#14, #15, #23, #24]. The difficulties extended to participant communications with program staff, with some individuals requiring multiple reminders to submit updated weight and physical activity data [#4].

### Issues around data collection and management

Data collection emerged as another aspect impacting the achievement and maintenance of CDC recognition. Maintaining accurate data records was highlighted as a barrier. Challenges included the database system utilized by one organization, which was cited as often slow in returning cleaned data needed for recognition submissions [#15]. One respondent emphasized the need for ongoing data support to facilitate data extraction and creation of reports [#23] and another noted the time-consuming nature of capturing and reporting data [#24].

### Program costs and financial barriers

Respondents reported financial challenges in maintaining recognition, particularly due to inadequate reimbursement rates. One program director noted that while grant funding helped sustain their program, it was not a long-term solution [#2]. Another respondent highlighted issues with pending recognition, which prevented payments from insurance companies [#3]. The struggle with self-funding was evident, as one health integration executive mentioned difficulties with enrollment for self-pay participants [#14], and a practice manager explained that their for-profit status limited grant opportunities, forcing them to cover costs out-of-pocket and ultimately halting the program [#25]. High employee turnover and resource shortages further complicated the financial sustainability of the program [#21].

## Theme III. Strategies to address challenges in maintaining recognition

Respondents were asked about strategies their organizations used to potentially address challenges. Responses indicated organizations (1) provided additional support to participants to enhance retention in the program; (2) increased referrals and recruitment; and (3) relied on partnerships with other organizations to support program delivery.

### Additional support to participants to enhance retention

Providing additional support to participants emerged as a key strategy. Respondents discussed personalized strategies to enhance participant engagement, such as additional sessions for those who missed the previous one, enrollment in additional cohorts, and individual follow-ups with lifestyle coaches [#1, #4, #5, #6]. Efforts to connect participants with resources and overcome barriers, such as transportation issues, were common initiatives, including providing bus passes and gas gift cards [#2, #7, #9, #11, #27]. Additionally, delivering the program in community-based locations, and including evening classes were discussed as strategies to enhance accessibility and increase retention rates [#17, #19, #24]. One organization employed diverse methods to increase retention, including raffles, individual goal-tracking support, and reminder calls [#23].

### Increasing referrals and recruitment

Organizations employed diverse strategies to increase referrals and recruitment for the program. A community health supervisor mentioned participating in community events to garner referrals [#3]. Another common approach involved ongoing engagement with the organization’s leadership, healthcare partners, and other partners outside the organization to increase awareness of the program and improve referrals [#5, #8, #20]. Promoting the program through different forms of advertisements, such as emails, flyers, TV, and radio interviews, was also discussed as strategies employed [#12].

### Partnerships with other community organizations

Similar to the theme of achieving recognition, respondents emphasized that partnerships with community organizations helped them to overcome challenges around maintaining recognition. Collaborations with healthcare organizations, such as local community hospitals and federally qualified health centers, provided critical resources and support [#7]. Partnerships with state and local health departments enabled programs to “combine efforts, apply for larger pools of funding, and create networks” to address participant needs holistically [#11]. Faith-based and community groups also played a key role, with one program director emphasizing the importance of “strong ties to the community” [#6]. Additionally, partnerships with payers helped secure contracts and advocate for insurance coverage [#2].

## Theme IV. Enhancing CDC support

Respondents reported that the CDC can better support organizations that deliver the National DPP by (1) adapting program requirements needed to achieve recognition; (2) continuing and enhancing the provision of resources and training; (3) improving funding and insurance reimbursement models; and (4) increasing awareness of prediabetes and the National DPP. In addition, while not prompted, some respondents demonstrated appreciation for the CDC support, which we include below.

### Program adaptations and changes in requirements for recognition

Respondents highlighted the need for flexibility in the curriculum to better accommodate diverse populations, suggesting that a one-size-fits-all approach may not be effective for diverse populations [#1, #9]. One organization also recommended adjustments to data collection targets (e.g., participant body weight), noting that modifications could help address higher dropout rates among vulnerable groups and improve data reporting [#5]. Additionally, a respondent suggested reducing the number of reporting deliverables required for program recognition [#21]. There was also a call for shorter program duration, with two respondents noting that this could increase participant engagement and retention [#7, #19].

### Resources and training

Respondents underscored the need for the CDC to continue providing general resources, webinars, and training related to National DPP delivery [#4]. One organization recommended ongoing access to online content and supplemental resources to enhance the curriculum, along with continuous training opportunities [#6]. Specific calls for more targeted training were made, including guidance on data management such as data entry and access, and the implementation of regular question-and-answer (Q&A) sessions to address ongoing questions and challenges [#18]. Additionally, there was a request for tailored recruitment strategies for diverse rural populations, as these have been identified as significant challenges [#15].

### Improving funding and insurance reimbursement models

Respondents underscored the challenges posed by insufficient funding for program delivery. While acknowledging the CDC's ongoing efforts related to the program delivery, they also expressed the importance of continued advocacy for higher insurance reimbursement rates and universal coverage for the program [#2, #24]. One respondent raised concerns about relying solely on insurance payments to cover the program, which may still fall short of covering program costs [#11].

### Increasing awareness of prediabetes and of the National DPP

Respondents highlighted that increasing awareness about prediabetes and the National DPP would improve recruitment and enrollment efforts. This included not only increasing awareness of the program among the medical and healthcare community [#8, #11], but also connecting potential participants to organizations delivering the program [#20].

### CDC is supportive

Respondents expressed gratitude for the CDC’s assistance, particularly in data review, highlighting the CDC’s supportiveness in providing quick and constructive feedback during the program delivery [#2, #13, #26]. Individualized meetings for report reviews and feedback further underscored the level of support received [#13]. One respondent expressed a desire to maintain this supportive relationship [#16].

## Discussion

This cross-sectional descriptive study aimed to provide qualitative data into the barriers and facilitators in achieving and maintaining CDC recognition to deliver the National DPP. The personnel from 27 National DPP organizations responded to a web-based questionnaire with open-ended questions. Four main themes emerged from the study, including (I) factors influencing achieving and maintaining CDC recognition, (II) challenges to achieving and maintaining recognition, (III) strategies for overcoming these challenges, and (IV) suggestions for improving CDC support. These themes provided information associated with barriers and facilitators to achieving and maintaining CDC recognition for the National DPP lifestyle change program. Our findings provide insights to inform practice and policy-level changes to support the scalability and sustainability of the National DPP from an organizational perspective.

From themes I, II, and IV, funding emerged as both a facilitator and barrier in achieving and maintaining CDC recognition. Adequate funding is crucial for implementing and sustaining lifestyle change programs, supporting key elements such as staffing, participant recruitment and retention (e.g., costs used to cover transportation), and program resources (e.g., maintaining facilities and providing digital tools for virtual program delivery). Our findings indicate that current funding models, which rely on organizational support, external grants, or insurance reimbursement, are often inadequate and difficult to sustain. Previous research corroborates our findings, showing that external grants are associated with the sustainability of National DPP lifestyle change programs, though they are frequently time-limited or insufficient to cover the full costs of program delivery [29, 30]. Moreover, the reimbursement models provided by public insurance, such as Medicare and Medicaid, have been described by our respondents and in the literature as often insufficient to cover the full range of costs associated with program delivery, particularly in areas requiring additional program participant support [31–33]. Further research is needed to quantify the full costs of delivering the National DPP, considering the need for diverse teams, comprehensive participant support, and other essential factors such as additional costs related to the recruitment and retention of participants, staff training and development, program materials and resources, technology infrastructure for virtual delivery, and ongoing evaluation and monitoring of program effectiveness [34]. Addressing these gaps will require building capacity among National DPP organizations and developing sustainable funding models that ensure the program’s long-term success, particularly in socially vulnerable communities.

Participant recruitment and retention emerged as significant challenges for maintaining CDC recognition based on themes II and III, with organizations adopting various strategies to address these issues. This finding aligns with previous research, which has identified recruitment and retention as major obstacles in delivering the National DPP, particularly among socioeconomically disadvantaged and racial and ethnic minority groups [14–16, 25, 34, 35]. These difficulties are often rooted in structural and social determinants of health, such as limited access to healthcare, financial constraints, competing priorities such as work and caregiving responsibilities, and structural racism. These factors contribute to systemic barriers that disproportionately affect socioeconomically disadvantaged and racial and ethnic minority groups, potentially hindering their ability to access and remain engaged in programs like the National DPP [36]. Insufficient recruitment and low retention, likely influenced by these structural and social determinants, coupled with participants failing to meet required outcomes, can undermine organizations’ ability to maintain CDC recognition. This is critical, as CDC recognition directly impacts eligibility for Medicare, Medicaid, and other insurance reimbursements, which are contingent upon participants achieving specific attendance and weight loss goals [9]. The financial strain imposed by these challenges can lead organizations to pause program delivery or withdraw from the National DPP altogether—a concern echoed by respondents in our study and stakeholders in previous qualitative research [33]. Notably, over 3,000 organizations were listed as providers of the National DPP between 2012 and 2019 [10]. By the start of our data collection in 2022, this number had dropped to just over 2,000, and as of October 2024, only about 1,500 organizations are providing the program [37]. In light of these challenges, our respondents highlighted several effective strategies, including makeup sessions, individualized follow-ups, transportation incentives, and community-based program delivery. These personalized approaches are consistent with practices reported by several National DPP sites in California and New York [38–40]. However, while these strategies enhance participant engagement and retention, they may also lead to increased operational costs. This underscores the need to potentially integrate these additional expenses into reimbursement models, including the adoption of risk-adjusted payment models that account for the costs of additional supportive strategies associated with the issues highlighted above [34].

Theme IV provided information about the need for modifications and greater flexibility in CDC recognition requirements to support the long-term sustainability of the program. Respondents suggested that adjusting the curriculum to better accommodate diverse populations and reducing the number of required reporting deliverables could improve program delivery. Flexibility in data collection targets, such as reducing the 5% target for participant body weight, was also recommended to address dropout rates among vulnerable groups and improve reporting accuracy, a recommendation echoed by other National DPP organizations in a previously published study [31]. Importantly, the CDC has recently amended its requirement for full recognition, now allowing a weight loss target of 4% if other metrics, such as minimum attendance and physical activity minutes, are met [8]. This change illustrates CDC’s willingness to adapt its policies to better align with the needs of organizations, potentially enhancing their capacity to maintain recognition and sustain the program. Additionally, from Theme IV, respondents highlighted the importance of raising awareness of prediabetes and the National DPP among healthcare professionals and the general public to increase referral rates and participation. Currently, only about 15% of individuals with prediabetes in the U.S. are aware of their status [41]. Enhancing this awareness is crucial, as individuals who are aware of their prediabetes status are significantly more likely to engage in lifestyle changes and enroll in lifestyle change programs, such as the National DPP [34, 42–44].

Moreover, respondents underscored the need for ongoing and expanded CDC support in several critical areas, including the sharing of best practices for DPP delivery, improved data management systems, and the collaboration among the National DPP organizations, as described under Themes III and IV. Continuous engagement with the CDC, especially through individualized feedback and tailored support, was appreciated and regarded as vital in creating a supportive environment for National DPP organizations. Additionally, respondents highlighted the need for more targeted training and resources to help organizations address specific challenges, particularly in the areas of data management, participant retention, and program delivery. Furthermore, expanding access to technical assistance and peer-learning opportunities could foster greater knowledge exchange and innovation among delivery organizations. According to some organizations, strengthening CDC support in these areas is essential for improving the overall effectiveness, scalability, and sustainability of the program, particularly as it expands to serve more diverse and historically underserved populations.

The barriers and facilitators identified in this study align with broader recommendations from the American Diabetes Association (ADA) Standards of Care and the World Health Organization (WHO) Global Action Plan for the prevention and control of non-communicable diseases. The ADA emphasizes the importance of accessibility to evidence-based diabetes prevention programs and the importance of tailoring interventions for social context. Our findings, which highlight challenges in participant recruitment and retention, are consistent with ADA’s calls for assessing and addressing social determinants to ensure equitable access to prevention programs [45]. Similarly, the WHO’s Global Action Plan underscores the need to improve the accessibility of healthcare interventions, particularly for vulnerable populations, through policies that promote sustainable, community-centered interventions [46]. Our study’s findings about tailoring the National DPP to meet the needs of socially vulnerable communities resonates with these international frameworks, as both the ADA and the WHO advocate for the customization of health programs to address local needs and circumstances. In particular, the WHO’s emphasis on multisectoral approaches to disease prevention, integrating community and healthcare resources [46], aligns with the strategies suggested by respondents in this study, such as partnerships between community organizations and individualized support. These parallels highlight that the challenges and opportunities for improving the National DPP are not unique to the U.S. but reflect global priorities for addressing chronic disease prevention in vulnerable populations.

Our study has several limitations. The voluntary nature of participation may have introduced selection bias, potentially overrepresenting organizations experiencing more challenges or those with more resources. This is particularly relevant as we sampled organizations in disadvantaged areas. The qualitative design limits the sample’s representativeness, possibly not reflecting the broader population of all National DPP organizations. However, we aimed to provide in-depth insights rather than comprehensive nationwide data. Similarly, while we aimed for 20–25 completed questionnaires to potentially achieve code saturation, as described in the methods, this was an estimated sample size, and we do not claim certainty about having achieved full data saturation. Additionally, we did not account for diversity in the type and size of organizations or contextual factors such as local policies and community health initiatives, which could impact an organization’s ability to achieve and maintain CDC recognition. These limitations suggest areas for future research to enhance the generalizability and applicability of our findings.

Future research should address several key areas based on this study’s insights. First, a comprehensive quantitative analysis across a broader and more diverse sample of National DPP organizations is needed to validate these findings and identify generalizable predictors of successful program delivery and CDC recognition maintenance. Investigating the long-term impact of various funding models and reimbursement strategies on the sustainability of the National DPP, especially in socially vulnerable communities, can inform policy decisions. Additionally, examining the role of local and state policies and community initiatives in supporting or hindering the National DPP delivery could identify contextual factors needed for tailoring interventions to specific environments. Because this pilot study provides preliminary insights into barriers and facilitators, policy recommendations should be considered cautiously and within the study’s limitations. Based on our findings, policymakers could focus on enhancing access to care and addressing social determinants of health to support National DPP implementation, but larger, confirmatory studies are necessary to establish a robust evidence base for systemic changes. For example, funding mechanisms to reduce financial barriers and policies that promote interdisciplinary care models may help address the challenges faced by socially vulnerable communities. Finally, studies focusing on the integration of technology (e.g., electronic devices that can deliver the data automatically and AI) for data management could provide insights for reducing organizational burden and enhancing the National DPP’s efficiency.

## Conclusions

In conclusion, our study highlights key factors influencing the achievement and maintenance of CDC recognition for delivering the National DPP lifestyle change program. The availability of resources, strategic partnerships, and adequate funding emerged as critical facilitators, while challenges in participant recruitment, retention, and meeting program outcomes were significant barriers. Although organizations have implemented strategies to address these barriers, these efforts often increase operational costs. To ensure the scalability and sustainability of the National DPP, future efforts could focus on more flexible recognition requirements, improved funding, and reimbursement models that consider these additional costs, as well as enhanced training and technical assistance for program delivery. By addressing these considerations, policymakers and stakeholders can better support organizations in delivering effective and sustainable National DPP interventions, especially in socially vulnerable communities.

## Supplementary Information

Supplementary Material 1.

## Data Availability

Data is provided within the supplementary information file.

## Abbreviations

ADA: American Diabetes Association
ATSDR: Agency for Toxic Substances and Disease Registry
CDC: Centers for Disease Control and Prevention
HbA1c: Hemoglobin A1c
National DPP: National Diabetes Prevention Program
SRQR: Standard for Reporting Qualitative Research
SVI: Social Vulnerability Index
WHO: The World Health Organization
