# Supplementary material for: Navigating CDC recognition for the National DPP in socially vulnerable communities: barriers, facilitators, and recommendations
Source: Arch Public Health. 2025 Mar 3;83:57. doi: 10.1186/s13690-025-01532-4 (PMC11874788; doi:10.1186/s13690-025-01532-4)
Supplement: Supplementary file 1 — Supplementary Material 1. [file 13690_2025_1532_MOESM1_ESM.docx]

**Supplementary Material**

**Navigating CDC Recognition for the National DPP in Socially Vulnerable Communities:** **Barriers, Facilitators, and Recommendations**

**Corresponding author:** Taynara Formagini, 9500 Gilman Dr. La Jolla, CA 92093. Email: tformagini@health.ucsd.edu

1. **Sample of open-ended questions included in the questionnaire**

| Q1. | Name of organization. |
| --- | --- |
| Q2. | What is your position within the organization? |
| Q3. | Think back to when you achieved CDC recognition to deliver the National DPP. Why do you think your organization was able to achieve CDC recognition? (e.g., resources, leadership, team designated to develop program, etc.) |
| Q4. | What were the challenges your organization encountered to achieve CDC recognition? If no challenges were faced, please write “N/A.” |
| Q5. | How were these challenges addressed? Was that successful? Please explain. |
| Q6. | What are the challenges that your organization is currently facing in maintaining CDC recognition? |
| Q7. | Why do you believe your organization is able to maintain CDC recognition? |
| Q8. | In your opinion, how can the CDC better help your organization maintain recognition so you can continue to deliver the National DPP? |
| Q9. | Now think about the delivery of the program. Why do you think your organization was able to start delivering the DPP lifestyle change program? |
| Q10. | What were the challenges your organization encountered when the program was first implemented? If no challenges were faced, please write “N/A.” |
| Q11. | What are the challenges that your organization is currently facing in delivering the program? If no challenges, please write “N/A.” |
| Q12. | How is your organization managing or addressing these challenges? |
| Q13. | Why do you believe your organization is able to continue to deliver the National DPP? |

**Note:** For questions Q9 to Q11, we included quotes from participants only when the themes addressed in their responses were pertinent to the achievement and maintenance of CDC recognition. Although these questions focused on program delivery in general, we understand that some of the challenges and successes in delivering the National DPP are closely related to the organization's ability to achieve and maintain CDC recognition. Therefore, only those responses that provided insights relevant to the recognition process were incorporated into the manuscript.

1. **Themes and supporting participants’ quotes**

| **Theme I. Factors associated with achieving and maintaining CDC recognition** | |
| --- | --- |
| Availability of resources and funding sources | *… the resources and leadership training provided by the local department of health. [#4, program director]*  *We had strong admin support, resources (people) available to work through the process, and a team approach between the [university] Health Hub, [university] School of Pharmacy, and [university] Health System Dietetic Internship in order to kick things off. Grant support through the [state] Department of Health was also imperative. [#5, educator, dietitian]*  *We achieved CDC recognition for NDPP we were able to do so because we had a funding source that allowed us access to resources to engage the community. [#7, health education coordinator]*  *We are well supported by our parent organizations, which are two large healthcare systems in our county. [#10, program manager]*  *Covering the cost of staff time sufficiently was a barrier that was minimized through grant funding. [#11, director of community health]*  *Resources (fitness center location with community benefit funding from the hospital). [#12, DPP coordinator, lifestyle coach]*  *Also, our staff were given time during the workday to attend class. [#19, chronic disease prevention program manager]* |
| Team commitment to deliver the program | *We also had a strong group of CHW's that were trained and certified to help with referrals to the program. [#7, health education coordinator]*  *…due to the certified lifestyle coaches' dedication to engaging with participants to keep them coming to classes and/or doing makeup classes. [#9, lead outpatient registered dietitian]*  *We have high-quality coaches, all with a minimum of a bachelor’s degree in a health-related field and that are trained in behavior change modification strategies. [#10, program manager]*  *Leadership (Lifestyle Coaches are Health Care Professionals (BS, MS degrees). [#12, DPP coordinator, lifestyle coach]*  *Lifestyle Coaches were key. This is a one-year program that requires committed investment in facilitation and follow-up with program participants. It was important for coaches to develop trusting relationships to maintain retention. [#13, community health supervisor]*  *We had a bilingual team, passionate about serving the community. We had CHWs, staff and med students. [#24, program director]*  *We had the appropriate staff to deliver the program (several health educators) and knowledge of how the program worked at the time. [#25, practice manager]*  *Our CEO is very much about wellness and preventative medicine and wanted to do right by our participants and community. [#26, customer services representative]* |
| Partnerships with community organizations | *We developed the [name of the program] Initiative to reach the African-American community with National DPP through outreach to the Faith community. All of the examples [help us to] achieve recognition, most especially the partnerships with the Faith and community organizations. [#6, program director]*  *[the university personnel] have access to local communities with which they work and the skills to set up and deliver programs. [#8, community outreach specialist]*  *I feel that statewide partnerships were valuable, and provided us with access to tools, resources, and funding that made this possible […]. Working with partners to offload some of the burden of billing allows us to build a case for support for our leadership as well as sustainability at minimal cost to our organization. We are very grateful for the community partners that make this program possible. [ #11, director of community health]* |
| **Theme II. Challenges in achieving and maintaining CDC recognition** | |
| Recruitment and retention of participants | *Securing participants to host the number of cohorts needed to meet the data requirements. [#1, division manager]*  *Barriers to participation related to social determinants of health (lack of transportation, access to affordable healthy food, etc.)[..] [#2, program director]*  *My challenges were getting referrals for participation into the program. [#3, lifestyle coach]*  *Participation rate decreased during the monthly sessions. [#4, program director]*  *Sustaining participation amongst participants, referrals to the program to host additional cohorts. Lack of participant supplied data consistently amongst virtual attendees. [#5, educator, dietitian]*  *Participant retention is always a challenge. Motivation of individuals to embrace and employ lifestyle change. […] [#6, program director]*  *Challenges we faced in order to achieve recognition were participant retention. Many participants didn't want to participate in a yearlong DPP program and the few that did would drop after a few weeks of participation […] Recruiting participants and participant retention is a challenge we are still facing in order to maintain the CDC recognition. [#7, health education coordinator]*  *The biggest challenge seems to be recruitment and reaching the medical community to refer to the program. [#8, community outreach specialist]*  *It's challenging to recruit people into the program due to the time commitment required up-front. [#10, program manager]*  *Participant retention will always be a challenge - participants encounter many barriers throughout their journey and helping them overcome those is time-consuming (but necessary). This can impact program outcomes and retention [#11, director of community health]*  *Assuring that we have at least 5 people finishing each cohort (so far have been successful with this for our last 4 cohorts. Really only had difficulty with this trying to recruit during the middle of the pandemic. [ #12, DPP coordinator, lifestyle coach]*  *Obtaining participants for the program. [#16, health educator]*    *People are dropping out after the first 6 months. [#17, coordinator]*  *Challenges our organization encountered when achieving CDC recognition was that sometimes our [participants] had competing interests, such as work-related responsibilities, which made it difficult for them to attend every class. [#19, chronic disease prevention program manager]*  *It is hard to get enough people to participate [#22, membership director]*  *We were not able to continue to deliver the DPP because we could not get enough people to commit to the program, in other words, the enrollment was not high enough. This convinced our organization's administration that the program was not worth continuing. [#26, customer services representative]*  *Patient participation and keeping patients motivated throughout the year long program. [ #27, nurse educator]* |
| Participants not meeting program outcomes | *Challenges have included […] participants not meeting the requirements to be considered a success… [#1, division manager]*  *A couple of participants are not very responsive and required multiple reminders to get their updated weight and minutes of physical activity. [#4, program director]*  *Participant weight loss [#14, health integration executive]*  *We are currently facing challenges in maintaining recognition because we've been relying on the weight loss/physical activity standards for recognition, but not all participants have been reaching this standard--particularly with the onset of the pandemic and the decrease in physical activity across many populations. [#15, MDPP regional coordinator, public health educator]*  *Challenges with program retention and weight loss achieved [#23, health equity program director]*  *It was very challenging to meet the weight loss and attendance rates. [#24, director]*  *[We were] unable to meet the requirements for participant outcomes. [ #27, nurse educator]* |
| Issues around data collection and management | *The data component. We had to ensure we maintained accurate data records. When the standards changed in 2021, that extended our Preliminary status, and we were unable to receive full recognition. [#13, community health supervisor]*  *Additionally, the database system our state contracts with is oftentimes slow with returning the cleaned data we need to submit for recognition. [#15, MDPP regional coordinator, public health educator]*  *Ongoing data support to pull data and create reports; [#23, health equity program director]*  *Capturing and reporting data was time-consuming for the program coordinator. [#24, director]* |
| Program costs and financial barriers | *Inadequate reimbursement rates and lack of universal coverage for this program!! We have managed to sustain our programs thanks to grant funding, but we won't be able to do that forever. [#2, program director]*  *I am not getting full recognition status from Medicare and my organization is not able to receive any payments from various insurance company due to my pending recognition. [#3, lifestyle coach]*  *Participant enrollment for those who have to self-pay [#14, health integration executive]*  *It may be hard to maintain CDC recognition due to the high turnover of employees and now the lack of resources to run the classes. [#21, health education manager]*  *Financially, it just didn't make sense. As a private practice, we were paying out-of-pocket to try to get this program up and going. […] As a for-profit, we aren't eligible for too many grants. We could not afford to continue to pay staff to be able to reach the Medicare billing. [#25, practice manager]* |
| **Theme III. Strategies to address challenges in maintaining recognition** | |
| Additional support to participants to enhance retention | *Facilitators work to promote and provide support to participants in between sessions. [#1, division manager]*  *We hired and trained a Community Health Worker who connected participants with resources to reduce these barriers. We now include funding for wraparound services with every DPP grant we submit. [#2, program director]*  *I put a lot of effort into communicating with participants and made sure to provide as much support as possible to help them maintain their goals for this program. […] When a participant missed a class, I made sure to communicate with the person and provided make-up sessions. I also enrolled participants to another national program, Walk with Ease, in the period of monthly sessions to help participants to stay engaged. These two strategies were pretty helpful. They helped the participants to hold themselves more accountable. [#4, program director]*  *[the staff] partner up with our participants to be their personal Lifestyle Coach to follow up with weekly after class to engage, answer questions, and gather information. Not all participants have expressed interest. [#5, educator, dietitian]*  *We addressed retention by allowing our lifestyle coaches to hold as many sessions, beyond the 26 modules, as were mutually agreed upon between them and the participants. This aided in retention. We also provided physical activity to all of our sessions. [#6, program director]*  *We are trying to access barriers in participation, for example, transportation. We have tried providing centralized locations where the program is offered, bus passes, and gas gift cards. However, we still struggle with participant retention. [#7, health education coordinator]*  *We are looking into stipends so we can provide metrocards for easier access to come for the in-person classes. [#9, lead outpatient registered dietitian]*  *We take a truly wraparound approach for all participants, meeting them where they're at and helping them overcome challenges […] We also have a Community Health Navigator on staff who works with participants on overcoming barriers to participation. [#11, director of community health]*  *We offer the program free of charge and either during or after the workday. [#17, coordinator]*  *We also started a community cohort and offered classes after 5pm. [#19, chronic disease prevention program manager]*  *We increased retention in a few ways-instituted a raffle program where participants received a raffle ticket for each class attended, offered individual goal tracking support, conducted frequent reminder calls, instituted a "buddy" system which increased accountability among participants [#23, health equity program director]*  *Offering the program at community-based locations and implementing innovative engaging strategies helped with retention. However, many of our participants had irregular job schedules conflicting with the class, and others didn’t have reliable transportation, which impacted attendance. [#24, director]*  *We provided incentives to participants to keep them engaged and participating throughout the year [#27, nurse educator]* |
| Increase referrals and recruitment | *I participated in community events to get referrals. It was not successful as the attendees wanted community resources and social needs. [#3, lifestyle coach]*  *We continue to engage with our leadership and other healthcare partners to assist in community recruitment through various channels. Success has been variable. [#5, educator, dietitian]*  *The program website provides a way for interested members of the public to sign up for the program. Referrals to the websites come mostly from people searching the internet for help after a diagnosis of prediabetes. Reaching out to state employees at universities has proved successful [#8, community outreach specialist]*  *We put a lot of effort into recruitment through our own advertising (Emails, flyers, community tabling, TV, radio interviews). We do have some referrals from our hospital, and we do get participants from our fitness center, which is in the same building as us.[ #12, DPP coordinator, lifestyle coach]*  *[We] reached out to providers in our medical system to increase awareness of the program. [#20, director of the diabetes education center]* |
| Partnerships with community organizations | *We are partnering directly with payers in our area to either establish contracts or to advocate for coverage. [#2, program director]*  *Our partners are committed to providing services like the DPP to the community in which the facility resides. We see the value added and the impact it can make on those who make it through the entire program. [#5, educator, dietitian]*  *Strong ties to the community — both Faith and otherwise. [#6, program director]*  *We are able to maintain recognition thanks to multiple partnerships in the community. We have strong relationships with the local community hospital as well as the local federal Qualified Health Centers. [#7, health education coordinator]*  *We have great relationships with our state health departments, as well as local city/county health care organizations, which allows us to combine efforts, apply for larger pools of funding, and create networks. We take a truly wraparound approach for all participants, meeting them where they're at and helping them overcome challenges. [#11, director of community health]* |
| **Theme IV. Enhancing CDC Support** | |
| Program adaptations and changes in requirements for recognition | *Understanding that a curriculum is not one size fits all and to be flexible on the requirements [#1, division manager]*  *Adjustments to the data collection targets- especially with higher drop-out rates amongst our most vulnerable populations for such a long program. [#5, educator, dietitian]*  *It would be ideal if the CDC could design a shorter curriculum that can be used in place of the Prevent Type 2 curriculum. There is huge interest in the program but once people learn that its a yearlong program they no longer want to participate. [#7, health education coordinator]*  *Get rid of the weight requirements for enrollment, the weigh-ins, and the weight loss requirements. Make it more weight inclusive. Better to go based on [physical activity] minutes and a1c's to measure health and success. [#9, lead outpatient registered dietitian]*  *I think the program could be shortened. Most participants reach their goal by the 6-month mark and no longer see the purpose of continuing the classes because they've accomplished what they set out to do. Also, I would recommend that the weekly weigh-ins would reduce to monthly or bi-weekly. [#19, chronic disease prevention program manager]*  *Lower the amount of reporting deliverables required to maintain recognition. [#21, health education manager]* |
| Resources and training | *Continue to provide general resources, webinars, and training for organizations that are conducting the program. [#4, program director]*  *Continue to provide the online content and resources to augment the curriculum. Also, by providing ongoing training opportunities. [#6, program director]*  *I would appreciate more tailored recommendations for recruitment amongst diverse rural populations. Recruiting and retention have come up as our biggest challenges to getting programs started. [#15, MDPP regional coordinator, public health educator]*  *Support us with training. Training on the data portion on how to enter, what to enter, how to access the data. I think a Q&A on a weekly basis may be helpful [#18, health education specialist]* |
| Improving funding and insurance reimbursement models | *This is less relevant to maintaining recognition, but any advocacy the CDC could do around advocating for better reimbursement rates and universal coverage of the program would be much appreciated! (I realize they are working hard at this already) [#2, program director]*  *Continued funding for these types of programs […] Sustainability is still tenuous - while reimbursements for Medicare/caid have improved, relying upon insurance payouts alone would not be sufficient to cover the cost of running the program. [#11, director of community health]*  *Improve funding models. We were unable to continue the program because of lack of funding. Payment model is inadequate and insufficient. [#24, membership director]* |
| Increasing awareness of prediabetes and of the National DPP | *Promoting the program in the medical community as a resource they can use to help their patients. Promoting HbA1c/blood glucose testing so that more people are aware that they have prediabetes. [#8, community outreach specialist]*  *Creating further awareness of prediabetes within the healthcare community and building bridges between these types of programs and healthcare providers […] Continuing to innovate in communication about these types of programs to eligible individuals [#11, director of community health]*  *Connect us with more participants who are interested in the year-long program [#20, director of the diabetes education center]* |
| CDC is supportive | *We have generally found the CDC to be incredibly supportive, particularly from a data review standpoint. [#2, program director]*  *CDC has been extremely helpful during the process. Their data collection may be tedious, but they offer quick and supportive feedback as needed. They have also met with me on an individual basis to review evaluation reports and provide feedback for the next cohort. Maintaining this level of support would be great. [#13, community health supervisor]*  *I feel that they are helping us with that right now. [#16, health educator]*  *I think the CDC and the Hispanic Federation were very supportive to us in delivering the DPP. Ultimately, the CEO made the decision to end it because we were not enrolling enough people into the program. [#26, customer services representative]* |
